# Evaluation of bacterial and fungal contamination of kitchens of Birjand University of Medical Sciences

**DOI:** 10.1186/s13104-019-4741-y

**Published:** 2019-10-28

**Authors:** Seyyedeh Masoomeh Rahimi, Maryam Ebrahimi, Behnam Barikbin, Tayebeh Zeinali

**Affiliations:** 1 Student Research Committee, Birjand University of Medical Sciences, Birjand, Iran; 2 Social Determinants of Health Research Center, Department of Environmental Health Engineering, School of Health, Birjand University of Medical Sciences, Birjand, Iran; 3 Infectious Diseases Research Center, School of Health, Birjand University of Medical Sciences, Birjand, Iran

**Keywords:** Microbial colony count, Fungal count, Foodborne diseases, Food Poisoning

## Abstract

**Objective:**

This study aims to evaluate the total bacterial and fungal count of tools, devices and surfaces of kitchens of the centers affiliated to Birjand University of Medical Sciences. In this study, 200 samples from four kitchens of Birjand University of Medical Sciences were obtained. After the preparation of serial dilutions, samples were cultured in plate count agar (PCA) plates and Sabouraud dextrose agar (SDA). After incubation at 37 and 25 °C for 24–48 and 72–96 h respectively, the microbial and fungal colonies were counted.

**Results:**

The mean bacterial and fungal count of kitchens was 7.7 * 10^7^ and 7.6 * 10^4^, respectively. The highest and lowest levels of bacterial contamination were related to tools/devices and cover of tools/work clothes and the highest and lowest levels of fungal count were related to forks and spoons and the tools and devices of the storage site. The rate of contamination in the kitchens of Birjand University of Medical Sciences was relatively high. Thus, serious, continuous and accurate monitoring of the units, training of people working in all stages of cooking and disinfection the tools and devices are essential for control and prevention of disease transmission.

## Introduction

There are no accurate statistics on the incidence of food poisoning and infections in developing countries as like as Iran, but undoubtedly due to inappropriate conditions of production, storage, distribution and consumption of food, and low level of public health education, the prevalence of food infections were higher in comparison with the developed countries [1, 2]. Based on the Gholammostafaei [3], the transmission of this disease by hand, food, and kitchen surfaces had been reported from 5 to 100%. Several reports have been indicated that food-borne diseases are a global problem [4, 5]. Contaminated food causes 1.5 billion cases of diarrhea in children annually, leading to more than 3 million deaths all over the world [3, 6, 7]. Considering the key role of the kitchen in transmitting pathogens to food and the role of food in transmitting the diseases to humans, we can realize its importance. Statistics suggest that kitchens and catering centers are among the most contaminated places compared to bathrooms and some toilet points or the equipment or devices that are used extensively, such as keys, cell phones, and so on [8].

In hospitals, the possibility of transmitting bacterial and fungal diseases can be multiplied in the absence of appropriate health conditions in the environment and equipment. By identifying these bacteria and fungi, we can find a way to eliminate them [9]. Nosocomial infections refer to the infections that have been caused as a result of host contact with agents causing the infection in the hospital. Most infections that occur 48 h after the patient’s hospitalization are considered as nosocomial infection [4, 10, 11]. Nosocomial infection is a problem that most hospitals face at the present time. The number and type of spores in the closed space of hospitals are sometimes the same as outside, and if the hospital and its internal environment produce fungal spores due to non-compliance with health standards, the infection will even increase. Infectious agents can be transmitted to patients through the contamination of medical equipment and physical environments. Statistics show that nosocomial infections affect 5% of hospitalized patients [11]. Moreover, the role of hospital food as a risk factor in the transmission of bacterial pathogens to the hospital environment and the onset of bacterial outbreaks has been emphasized in various reports. Annually, 1.8 million people die due to diarrhea diseases caused by contaminated food and drinking water in the world. In recent years, the number of nosocomial infections resisting treatment has increased in the world, and it seems that nosocomial infections increase in the future [3].

*Clostridium botulinum*, *Campylobacter*, *Escherichia coli*, *Salmonella*, and *Shigella* are among the bacterial pathogens transmitted by food. Since 1990 up to now, the three major food-borne bacteria groups (*Salmonella*, *Escherichia coli*, and *Campylobacter* species) have drawn the attention of many studies and food industries. However, the incidence of food-borne diseases has grown in developed countries. Several studies have examined the microbial contamination of hospital’s kitchens [1, 3, 12].

Despite extensive studies and efforts made to control infectious agents, especially in recent decades, human beings have not been able to eliminate these agents and the use of drugs such as broad-spectrum antibiotics, corticosteroids and immune-suppressions have been grown increasingly. Moreover, the emergence of immunosuppressive diseases such as AIDS and autoimmune diseases and the inappropriate conditions for hospitalization have led to a significant increase in hospital opportunistic infections [12]. Given the importance of bacterial outbreaks in Iran’s hospitals, especially in patients receiving immunosuppressive drugs or other treatments that are prone to bacterial infections, examining the role of hospital foods in the occurrence of these infections or the transmission of high-risk bacteria through them into hospital spaces have a particular importance [13]. As there is no information available on the health status of university kitchen devices, tools and surfaces, the aim of this study was to evaluate the total bacterial and fungal count of tools and surfaces of kitchen and cooking centers affiliated to Birjand University of Medical Sciences.

## Main text

### Materials and methods

#### Sample collection

A total of 200 samples were randomly taken from the tools, devices and surfaces in the kitchens of educational hospitals and other centers of the Birjand University of Medical Sciences since December 2017 to May 2018. Samples were taken from environment, tools and equipment such as floor, wall, sink, dishes, and refrigerators using a sterilized swab (wet method) in the morning shift. Accordingly, with regard to the surface, samples were taken from 50 cm^2^ of the considered surface using the template. Rinse method using Peptone water 0.1% was used to sample the items such as spoon and fork. The samples were immediately transferred to the laboratory at refrigerator conditions and were cultured within 2 h.

#### Total viable aerobic count (TVAC) of bacteria and fungi

Serial dilution was prepared from the samples. In order to assess the total bacterial count, the original sample and serial dilutions was pour-plated on plate count agar (PCA) and incubated at 37 °C for 24 to 48 h. With regard to the fungal load, the original sample and prepared dilutions were cultured in a Sabouraud dextrose agar (SDA) medium [14]. Then, the plates were incubated at 22–25 °C for 3 to 7 days. All the results were reported as cfu/ml.

#### Statistical analysis

The normal distribution of data was examined using Kolmogorov–Smirnov test [15]. As data did not have a normal distribution, nonparametric tests such as Kruskal–Wallis [16], were used to compare the mean bacterial and fungal loads in four sampling sites and Mann–Whitney test [17] was used for the pair wise comparison of sampling sites.

## Results

In the present study, there was a significant difference among four sampling sites in terms of mean bacterial count (p < 0.002) and fungal count (p < 0.026). In inter-group comparison, no significant difference was seen between code 1 and code 2 in terms of mean bacterial load (p > 0.05) and fungal load (p > 0.05). The mean bacterial load of code 1 was greater than that of code 3 (p < 0.007), but the mean of fungal contamination in these two sites was not different (p > 0.05). The mean of fungal contamination of code 1 was significantly less than that of code 4 (p < 0.006), but the mean bacterial load of these two was not significantly different (p > 0.05). The mean bacterial loads of code 2 and code 3 were significantly different (p < 0.001), but the mean of fungal contamination was not different (p > 0.05). Kitchen 2 and 4 did not show any significant difference in terms of bacterial and fungal contamination. The mean bacterial load (p < 0.002) and mean fungal contamination (p < 0.012) were significantly different in code 3 and code 4.

**Fig. 1:**
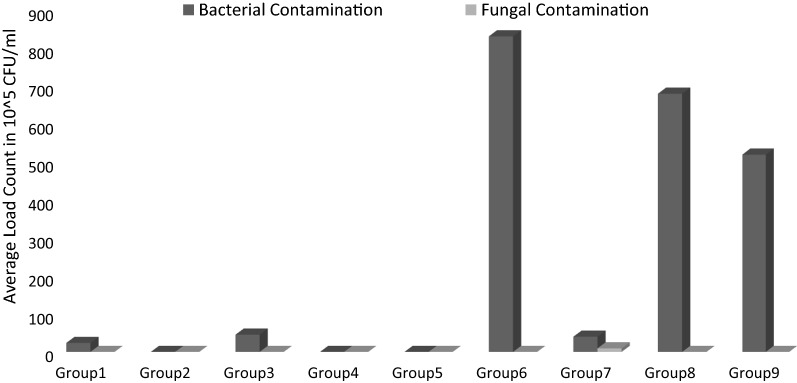
Comparison of the mean of bacterial and fungal contamination load in different groups

Figure 1 shows the mean bacterial load and fungal load in the nine specified groups.

**Fig. 2:**
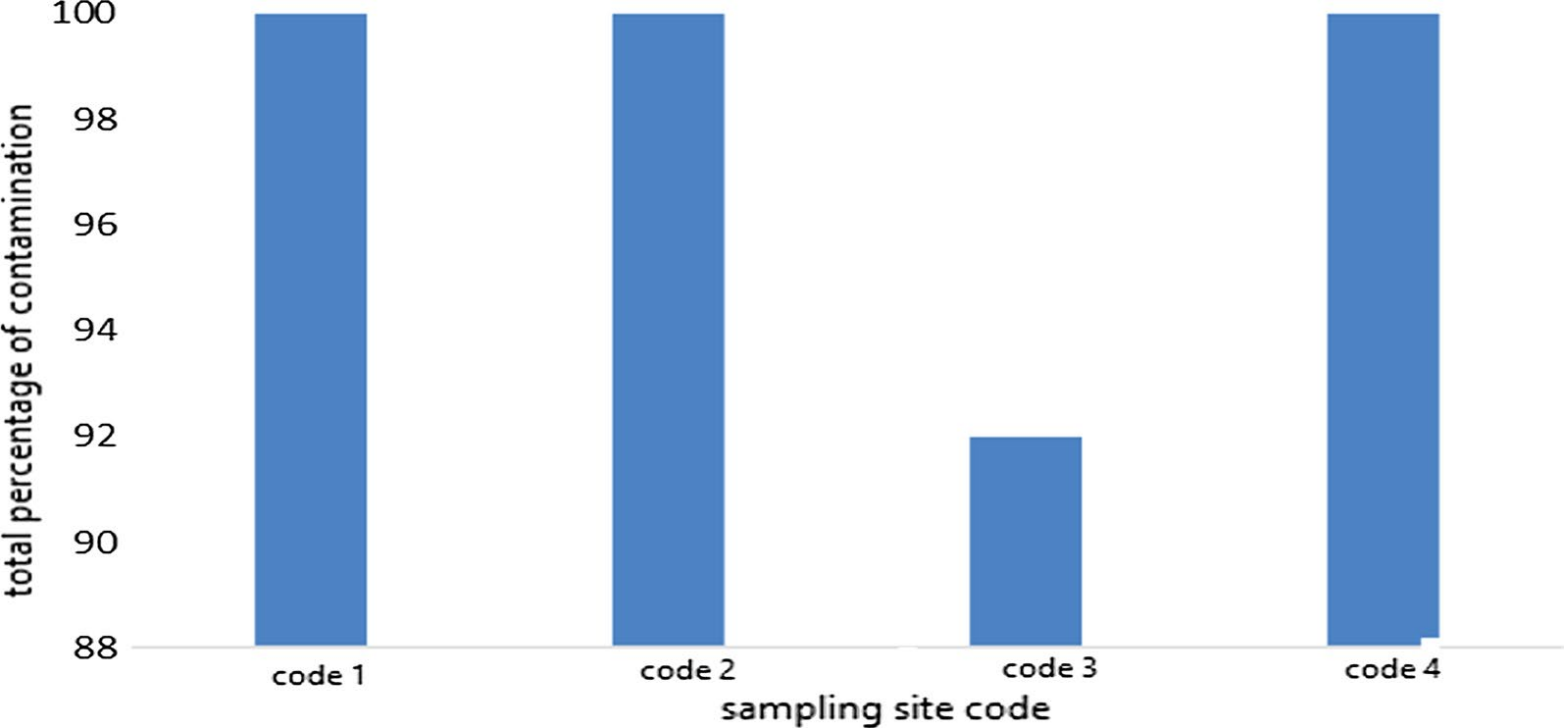
Frequency distribution of total contamination (bacteria and fungi) of sampled sites

The frequency distribution of contamination of the sampled sites is shown in Fig. 2. As shown in Fig. 2, three of the four sampled sites were contaminated by 100%.

Frequency distribution of bacterial and fungal contamination of different surfaces based on the standard plate count (between 30 and 300 colonies per plate, SPC) and estimated plate count (less than 30 colonies per plate or more than 300 colonies per plate, EsPC) are presented in Table 1.

**Table 1:** The frequency percentage of standard plate counts (SPC) and estimated plate counts (EsPC) of bacterial and fungal contamination in different groups

Group	Include	Type	0–30 (%)	30–300 (%)	> 300 (%)
1 (Kebab skewer)	All kebab skewers in kitchen	Bacteria	1 (8.3)	3 (25)	8 (66.7)
		Fungi	10 (83.3)	1 (8.3)	1 (8.3)
2 (food catering and distribution devices)	Food distribution containers, washed dishes, glass, tray, soup bowl, tea cup, teapot, sugar bowl, salt and pepper shakers	Bacteria	6 (50)	1 (8.3)	5 (41.7)
		Fungi	10 (83.3)	2 (16.7)	0 (0)
3 (cooking devices)	Chopper, iceplier, ladle, steam boiler, work knife, fish slice, pot, meat grinder, rice mixer, kitchen brush, stripper, grater, colander, ladle and kebab maker	Bacteria	4 (9.3)	8 (18.6)	31 (72.1)
		Fungi	31 (72.1)	6 (14)	6 (14)
4 (cover of tools and work clothes)	Apron, knob, meat grinder cover, chef’s gown, and cover of kebab maker	Bacteria	1 (12.5)	2 (25)	5 (62/5)
		Fungi	7 (87/5)	1 (12/5)	0 (0)
5 (food storage and transport tools)	food carrying containers, tray, trolley, bread basket, rice and wheat storage container	Bacteria	3 (18.8)	2 (12/5)	11 (68/8)
		Fungi	11 (68/8)	3 (18/8)	2 (12/5)
6 (equipment and devices)	Refrigerator, freezer, pastry maker, meat grinder, oven, kebab cooking device, food heater, balance, mixer, stripper	Bacteria	1 (2.5)	5 (12/5)	34 (85)
		Fungi	32 (80)	2 (5)	5 (15)
7 (spoon and fork)	spoon and fork and breakfast knife	Bacteria	3 (20)	0 (0)	12 (80)
		Fungi	9 (60)	1 (6/7)	5 (33/3)
8 (tools and devices of storage site)	Spoon holder, manual dishwasher holder, containers for holding skewers, cabinets, containers for storage of used tools and devices	Bacteria	0 (0)	5 (17.2)	24 (82.8)
		Fungi	25 (86.2)	4 (13.8)	0
9 (work table)	Meat chopping board, bread and vegetables chopping board, and breakfast table	Bacteria	1 (4)	6 (24)	18 (72)
		Fungi	6 (64)	5 (20)	4 (16)

## Discussion

The results of this study showed that 97.5% (Fig. 1) of the samples had bacterial contamination and 65.5% of the samples had fungal contamination. In the study conducted by Norozi [10], the kitchen and food products of centers affiliated to Iran University of Medical Sciences were examined. Out of 459 samples taken from food products, kitchen environment and food containers, 40.5% of cases had positive culture [10]. In the study conducted by Sanagu and Jouybari [11], the rate of kitchen contamination was 82.5%. In this study, the mean bacterial load of kitchens was 1.54 * 10^6^ cfu/cm^2^ [2]. In the study conducted by Heidari et al. [4], the microbial contamination of surfaces and air in different parts of the ShahidRajaei Hospital of Qazvin was examined. The research results showed that the most contaminated areas in the sampling were the surfaces of the kitchen area with 1.54* 10^6^cfu/cm^2^ [4]. In the study conducted by Gholammostafaei et al. [3], the frequency of bacteria isolated from the hospital kitchen cooking tools showed the highest contamination rate (40%). The highest percentage of microbial contamination in this study was found in devices with 50.2% [3]. In the study conducted by Norozi [10], the highest percentage of contamination was reported in the feeding table (87.5%). Moreover, in the study conducted by Borrusso in 2017, kitchen sink and kitchen sponges had the highest contamination (64%), which was not consistent with our results [18].

The reason for the high contamination of the devices can be attributed to the transfer of microorganisms directly from contaminated foods such as fresh vegetables, onions, potatoes, and carrots and non-completely washed meat to devices such as refrigerators. It might contaminate other foods, if not cleaned properly. In the present study, 20.8% of food distribution and catering devices and tools were non-contaminated and 79.2% were contaminated, which is somewhat consistent with the results of the study conducted by Norozi [10]. As kitchen tools are highly sensitive, they should be kept in very clean and low-humid conditions, because they have the ability to transmit many microbial agents to humans and, thus, have a great impact on their health [19, 20]. In terms of fungal contamination, the highest level of contamination was related to spoons and forks (98%). In the study conducted by Afsharyavari and Kambiz [12], the highest fungal contamination was related to trolley (63.6%). In the present study, comparing the contamination load of hospitals’ kitchens, the rate of the total microbial load was higher in hospital cooking site [12]. It can be a major cause of the high incidence of nosocomial infections. Non-adherence to the basic principles of the health by kitchen staff and their low level of information are key factors in this regard [21]. The results of this study showed that the infection rate of cooking devices in public hospitals was 85.7%, which was consistent with the results of the study conducted by Norozi, in which the rate of public hospital kitchen devices was reported 90%.

Unfortunately, in two cooking sites (50% of the sampling sites), the beetle was seen. In the study conducted by Norozi, beetles were seen 17.5%. Pests can easily transmit the contaminations to the food, so in order to prevent the entry of pests into such sites, proper poisons should be used in sites that are not directly related to the food [19, 22–24]. According to Table 1, in Groups 1, 3, 4, 5, 6, 7, 8 and 9, the highest percentage of bacterial contamination was related to above 300 colonies, while the highest percentage of fungal contamination was reported to below 30 colonies. Therefore, in these groups, due to the high percentage of bacterial contamination in the above 300 colonies column, the priority is to control bacterial contamination, which can be related to the inappropriate washing and disinfection. In Group 2, the highest percentage of bacterial and fungal contamination was related to below 30 colonies column, but it was scattered over the entire surface. It indicates that the transmission of the bacterial and fungal contamination related to the indirect and secondary transmission through the fungal spores at the wide area and in a closed environment such as cabinets or contaminated shelves.

## Conclusion

Finally, it is suggested that special attention to be paid to reduce the infections of cooking centers in critical sites such as educational, medical and welfare centers. Moreover, strictly and continuously monitoring and implementing the rules and regulations and environmental health rules in the process of preparing, cooking and distributing food in hospitals and kitchens and removing relevant defects is one of the cheapest and most appropriate preventive methods in this regard. Thus, relevant authorities and environmental inspectors should follow and implement it seriously.

## Limitations

One of the main limitations of the study was the limited number of kitchens associated with the university.

## Data Availability

All datasets in this study are available in reasonable request.

## Abbreviations

PCA: plate count agar
SDA: Sabouraud dextrose agar
TVAC: total viable aerobic count
SPC: standard plate counts
EsPC: estimated plate counts
